# Equity and Cancer Survival Among Veterans Health Administration Patients: A Systematic Review and Meta-Analysis

**DOI:** 10.1001/jamanetworkopen.2026.21585

**Published:** 2026-07-02

**Authors:** Alyssa Jasmine Bullard, Haley I. Tupper, Kevin Su, Lin Gu, Luca F. Valle, Isla P. Garraway, Donna L. Washington, Chloe E. Bird, Robert A. Winn, Stephen J. Freedland, Christina D. Williams, Drew Moghanaki

**Affiliations:** 1 Cooperative Studies Program Epidemiology Center-Durham, Durham Veterans Affairs Health Care System, Durham, North Carolina; 2 Division of General Surgery, Department of Surgery, David Geffen School of Medicine, University of California, Los Angeles; 3 Department of Radiation Oncology, David Geffen School of Medicine, University of California, Los Angeles; 4 VA Greater Los Angeles Healthcare System, Los Angeles, California; 5 Department of Urology, David Geffen School of Medicine, University of California, Los Angeles; 6 Center for the Study of Healthcare Innovation, Implementation & Policy, VA Greater Los Angeles Healthcare System, Los Angeles, California; 7 Department of Medicine, David Geffen School of Medicine, University of California, Los Angeles; 8 Center for Health Equity Research, Tufts Medical Center, Boston, Massachusetts; 9 Tufts University School of Medicine, Boston, Massachusetts; 10 The RAND Corporation, Santa Monica, California; 11 Virginia Commonwealth University Massey Comprehensive Cancer Center, Richmond; 12 Division of Pulmonary Disease and Critical Care Medicine, Virginia Commonwealth University School of Medicine, Richmond; 13 Durham VA Medical Center, Durham Veterans Affairs Health Care System, Durham, North Carolina; 14 Samuel Oschin Comprehensive Cancer Institute, Cedars-Sinai Medical Center, Los Angeles, California; 15 Department of Urology, Cedars-Sinai Medical Center, Los Angeles, California

## Abstract

**Importance:**

In the US, Black patients with cancer consistently experience worse survival compared to White patients, even after adjusting for age, sex, and disease stage. Whether these disparities exist among patients receiving care in the Veterans Health Administration (VHA), an integrated health system designed to provide near-equal access to care, remains uncertain.

**Objective:**

To evaluate whether overall survival (OS) and cancer-specific survival (CSS) differ between Black veterans and those with other race receiving cancer care through VHA.

**Data Sources:**

PubMed was searched from January 2015 through April 2022. Reference lists from identified studies were also reviewed.

**Study Selection:**

Studies of US veterans receiving cancer care through the VHA were included if they reported OS or CSS by race and provided hazard ratios (HRs). Dual independent rating of titles and abstracts was conducted for inclusion.

**Data Extraction and Synthesis:**

A random-effects model was used to pool effect sizes, the Paule-Mandel estimator was used to calculate the heterogeneity variance τ2, and Knapp-Hartung adjustments were used to calculate the confidence interval of the pooled effect. Review and meta-analysis was conducted using the Preferred Reporting Items for Systematic Reviews and Meta-Analysis (PRISMA) guideline.

**Main Outcomes and Measures:**

OS and CSS were compared between Black veterans and those who were not Black using pooled HRs.

**Results:**

Of 101 studies identified, 39 met inclusion criteria and 29 provided sufficient data for meta-analysis. The reported outcomes represented 603 256 veterans with cancer treated between 1983 and 2017, with a mean 29.0% (range, 8.9%-55.0%) of patients categorized as Black. All studies compared race by Black compared with White, except for 7 of 20 prostate cancer studies, which compared race by Black compared with non-Black. Black veterans were found to have better OS (HR, 0.93; 95% CI, 0.89-0.97) and CSS (HR, 0.94; 95% CI, 0.90-0.98). Survival advantages for Black veterans were observed across several cancer types, including bladder, laryngeal, lung, oropharyngeal, prostate, and plasma cell cancers. Between-study heterogeneity was low to moderate.

**Conclusions and Relevance:**

In this systematic review and meta-analysis of peer-reviewed publications reporting the outcomes of veterans receiving cancer care through VHA, survival outcomes were generally similar or better for Black compared with White or non-Black veterans. These findings suggest that integrated health care systems providing near-equitable access to comprehensive cancer care can substantially reduce or eliminate disparities in cancer outcomes.

## Key Points

**Question:**

Do disparities in cancer survival that are generally seen in the broader US population exist among Black veterans compared with veterans who are not Black receiving care through the Veterans Health Administration (VHA)?

**Findings:**

In this systematic review of 39 studies representing 603 256 veterans, including a meta-analysis of 29 studies with sufficient outcome data, Black veterans with cancer receiving care through the VHA had similar or better overall survival and cancer-specific survival compared with those whose race was categorized as either White or non-Black.

**Meaning:**

These results suggest that equity in outcomes for Black veterans with cancer is being achieved for those receiving care through the VHA.

## Teaser

This systematic review and meta-analysis of studies of US veterans receiving cancer care through the Veterans Health Administration compares outcomes for Black veterans compared with veterans from other racial groups.

## Introduction

Advances in cancer prevention, early detection, and treatment have led to significant declines in cancer mortality rates in the US over recent decades.^1^ Despite this progress, measurable differences exist among Black populations that are generally found to have lower rates of cancer survival for nearly every type of cancer, even after adjusting for sex, age, and disease stage at diagnosis.^1,2,3,4,5,6,7^ Meanwhile, multiple studies of veterans with cancer receiving care through VHA demonstrate comparable survival by race.

Key factors contributing to observed survival differences by race in the broader US population have been linked to social determinants of health (SDOH) such as lower socioeconomic status (SES) and reduced access to health care.^8,9^ Additional factors include disproportionately higher rates of environmental and occupational exposure to carcinogens associated with increased incidence, aggressiveness, and earlier onset of cancer.^10,11,12,13,14,15^ Individuals living in deprived neighborhoods, associated with non-White race, have been found to have more limited access to comprehensive health care, contributing to higher mortality rates compared with White patients once developing cancer.^8^

The Veterans Health Administration (VHA), the largest integrated health care system in the US, provides comprehensive cancer care for all eligible veterans and aims to provide equitable care regardless of race. In 2023, VHA served more than 9 million enrollees, representing approximately half of the 18.3 million veterans in the US.^16^ VHA care and coverage is relatively standardized, and beneficiaries can receive care at any VHA site in the country without insurance premiums and with minimal or no copayments.^17^ Eligible VHA beneficiaries with cancer have access to advanced cancer diagnostic services as well as medical, radiation, and surgical oncology care. In addition, VHA beneficiaries receive financial and nonfinancial assistance to ancillary services care such as transportation, housing, and telemedicine.^18^

Given that cancer survival outcomes rely on access to comprehensive care, and that VHA beneficiaries have near-equal access to health care regardless of race, it was hypothesized that Black veterans receiving their treatments through the VHA might have similar overall survival (OS) and cancer-specific survival (CSS) compared with veterans who were not Black.

## Methods

A systematic review of scientific articles published in PubMed was performed to investigate whether measurable differences in OS or CSS exist by race for veterans with cancer managed through the VHA. This study relied solely on publicly available data, which does not require institutional or regulatory approval.

### Search Strategy

We utilized the Preferred Reporting Items for Systematic Reviews and Meta-Analysis (PRISMA) reporting guideline to query PubMed (Medline) for peer-reviewed articles published from January 2015 through April 2022 to evaluate contemporary outcomes using the following search query: ((“Veteran*”[tw] OR “military personnel”[MeSH Terms]) AND (cancer*[tw]) AND (“racial”[tw] OR “ethnic*”[tw] OR “minority*”[tw] OR “race”[tw] OR “racial groups”[MeSH Terms]) AND (“survival”[tw] OR “mortality”[tw]) AND (“2015/01/01”[PDAT]: “2022/04/30”[PDAT])). Titles and abstracts were manually reviewed by 2 independent raters (A.J.B. and K.S.) to identify potentially relevant articles for full-text review.

Publications were included if they were original research reporting survival measurements by race for veterans with cancer who had received at least part of their care through VHA. Bibliographies of publications identified in the initial PubMed query that met inclusion criteria were reviewed to identify additional potentially eligible publications. Articles were excluded if found to be nonoriginal research, non–peer reviewed, not related to cancer care, lacking a cohort of patients managed by VHA, not related to cancer survival or mortality, or not reporting on survival or mortality by race and cancer type. Whenever consensus was not reached between the 2 raters, a third rater (C.D.W.) made the final determination. A free online tool was used to upload and organize the results of the literature search for systematic review.^19^

### Statistical Analysis

We compared differences in OS and CSS across eligible articles with a meta-analysis to estimate pooled hazard ratios (HR) and 95% CIs relying on available HRs whether adjusted or not. Race was categorized as Black, White, or non-Black. Articles not reporting the HR of OS or CSS comparing Black patients with White patients or Black patients with non-Black patients were excluded. A random-effects model was used to pool effect sizes, as we anticipated considerable between-study heterogeneity in terms of the types of cancers and treatments evaluated in the articles. The Paule-Mandel estimator was used to calculate the heterogeneity variance τ^2^.^20^ We used Knapp-Hartung adjustments to calculate the confidence interval of the pooled effect.^21^ We generated forest plots showing adjusted HRs to visualize OS and CSS comparisons of veterans identified as Black compared with White or non-Black. HRs for race were only extracted for comparisons between Black and White veterans or Black and non-Black veterans. Due to variability between articles in the meaning of the “other” racial category, HRs for this comparison group were excluded in the meta-analysis. To assess publication bias, funnel plots were produced to examine the association of precision errors with effect sizes of articles. Separate forest plots were generated for articles reporting outcomes for VHA patients with non-small cell lung cancer (NSCLC) and prostate cancer given they were more frequently reported. The analysis was conducted using the meta package version 6.2-1 of R software (R Project for Statistical Computing). Statistical significance was set at *P* < .05.

## Results

### Search Results

**Figure 1.**
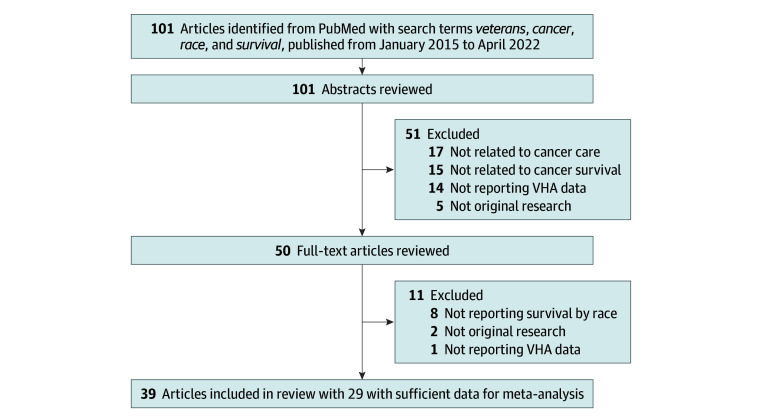
Systematic Review and Meta-Analysis Flow Diagram

The initial PubMed query identified 101 articles published from 2015 to 2022 with search terms related to veterans, cancer, race, and survival. Manual abstract reviews led to 50 eligible for full manuscript review after excluding 17 articles due to being unrelated to cancer care, 15 for not reporting cancer survival, 14 not reporting VHA data, and 5 not representing original research (Figure 1). Following full article review for the 50 remaining studies, 8 were excluded for not reporting survival by race, 2 not being original research, and 1 not reporting VHA data, resulting in 39 articles eligible for the systematic review. This led to the identification of outcomes for 603 261 veterans with cancer, ranging from 113 to 139 269 patients per study, and an average of approximately 17 000 per publication. No additional potentially eligible articles were identified after reviewing each manuscript’s references.

The majority of articles were retrospective cohort studies of veterans treated across the VHA. The number of VHA patients with cancer reported in each study ranged from 117 to 145 678 (mean [SD], 130 168 [714 567]; median [IQR], 3811 [837-18 466]). The percentage of veterans reported as Black ranged from 8.9% to 55.0% (mean [SD], 28.4% [10.2]; median [IQR], 27.4% [20.3%-34.3%]).

A total of 29 articles had sufficient data for the meta-analysis, including 20 (69%) focused on prostate cancer, 2 (7%) on NSCLC, 2 (7%) on pancreatic cancer, 1 (3%) on head and neck cancers, 1 (3%) on esophageal cancer, 1 (3%) on bladder cancer, 1 (3%) on breast cancer, and 1 (3%) on myeloma.

### Descriptive Analysis

Evaluations of OS and CSS reported by race for each cancer subtype are summarized below and in the Table. Of the 27 articles evaluating OS, the majority (17 articles [63%]) found similar rates by race, with 9 higher and 1 inferior for Black veterans. For the 17 articles evaluating CSS, 13 reported similar outcomes by race, with 3 higher and 1 inferior for Black veterans.

**Table.** Articles Included in Meta-Analysis

Source	Cancer type	Study period	Total veteran, No.	Black veterans, No. (%)	HR (95% CI)	
					OS	CSS
Kotha et al,^57^ 2022	Bladder	2000-2017	36 322	3250 (8.9)	0.99 (0.93-1.05)	0.97 (0.88-1.07)
Aggarwal et al,^58^ 2021	Breast	1998-2016	8864	2166 (24.4)	NA	NA
Nassri et al,^56^ 2018	Esophageal	2005-2010	122	25 (20.5)	NA	NA
Fullmer et al,^53^ 2020	Larynx	2000-2016	113	49 (43.4)	NA	NA
Ganti et al,^47^ 2016	Lung	1995-2003	7328	1267 (17.3)	0.92 (0.87-0.98)	NA
Heiden et al, 2022^48^	Lung	2006-2016	9842	1486 (15.1)	0.80 (0.73-0.88)	NA
Patel et al,^59^ 2020	Myeloma	1999-2014	3807	883 (23.2)	0.85 (0.78-0.93)	NA
Del Valle et al,^51^ 2022	Pancreas	2006-2017	919	168 (18.3)	1.10 (0.88-1.37)	NA
Toriola et al,^52^ 2020	Pancreas	1998-2013	3811	773 (20.3)	NA	NA
Anderson-Carter et al,^22^ 2019	Prostate	2000-2008	72 170	16 839 (23.3)	0.94 (0.90-0.98)	1.02 (0.95-1.10)
Chao et al,^23^ 2022	Prostate	2000-2010	1220	392 (32.1)	NA	NA
Courtney et al,^24^ 2021	Prostate	2001-2015	1007	330 (32.8)	1.15 (0.86-1.53)	1.21 (0.64-2.31)
Daskivich et al,^25^ 2015	Prostate	1998-2004	1122	505 (45.0)	NA	0.60 (0.28-01.26)
Deka et al,^27^ 2020	Prostate	2001-2015	8726	2280 (26.1)	1.0 (0.90-1.10)	1.2 (0.70-2.10)
Deka et al,^26^ 2020	Prostate	2001-2015	2857	835 (29.2)	1.26 (0.90-1.75)	NA
Dess et al,^28^ 2019	Prostate	1992-2013	3972	1513 (38.1)	1.05 (0.93-1.19)	0.85 (0.56-1.30)
George et al,^29^ 2021	Prostate	2014-2017	2910	787 (27.0)	0.67 (0.59-0.76)	NA
Howard et al,^31^ 2020	Prostate	1989-2016	547	301 (55.0)	NA	1.10 (0.42-2.97)
Howard et al,^30^ 2016	Prostate	2000-2013	233	80 (34.3)	0.92 (0.64-1.32)	NA
Khan et al,^32^ 2021	Prostate	1999-2013	4544	1352 (29.8)	NA	NA
Klebaner et al,^33^ 2021	Prostate	2004-2015	90 749	27 412 (30.2)	0.99 (0.96-1.04)	0.93 (0.85-1.02)
McKay et al,^34^ 2020	Prostate	2001-2015	31 131	9584 (30.8)	0.90 (0.85-0.95)	0.79 (0.69-0.92)
Moreira et al,^35^ 2017	Prostate	1983-2013	205	69 (33.7)	0.85 (0.59-1.22)	NA
Parikh et al,^36^ 2021	Prostate	2001-2013	40 412	10 605 (26.2)	0.93 (0.89-0.97)	NA
Patel et al,^37^ 2020	Prostate	2000-n/a	837	232 (27.7)	0.87 (0.73-1.04)	NA
Riviere et al,^38^ 2020	Prostate	2000-2015	60 035	18 201 (30.3)	0.84 (0.81-0.88)	0.85 (0.78-0.93)
Sarkar et al,^39^ 2020	Prostate	2001-2015	69 984	18 596 (26.6)	radiation, 0.94 (0.90-0.98); surgery, 1.02 (0.95-1.09)	radiation, 0.86 (0.77-0.97); surgery, 0.90 (0.76-1.07)
Sohlberg et al,^40^ 2020	Prostate	2000-2015	139 269	38 168 (27.4)	1.03 (1.01-1.05)	NA
Vidal et al,^41^ 2019	Prostate	1998-2015	595	241 (40.5)	0.94 (0.69-1.28)	0.92 (0.51-1.65)

Abbreviations: CSS, cancer-specific survival; HR, hazard ratio; OS, overall survival; NA, not applicable.

### Prostate Cancer

There were 25 articles reporting OS or CSS by race for veterans with localized or metastatic prostate cancer.^22,23,24,25,26,27,28,29,30,31,32,33,34,35,36,37,38,39,40,41,42,43,44,45,46^ All articles had a retrospective study design except for a single prospective phase III randomized clinical trial.^44^ Patients in these articles had been managed with either watchful waiting, active surveillance, radical prostatectomy, radiation therapy, or antineoplastic drugs, which included androgen deprivation and radionuclide therapies. The articles reported survival outcomes associated with the following potential prognostic factors: patient-related (race, obesity, depression, monocyte count, skeletal-related events), tumor-related (Gleason score, clinical and genomic risk classifiers), or noncancer therapies (metformin, statin, testosterone therapy).

**Figure 2.**
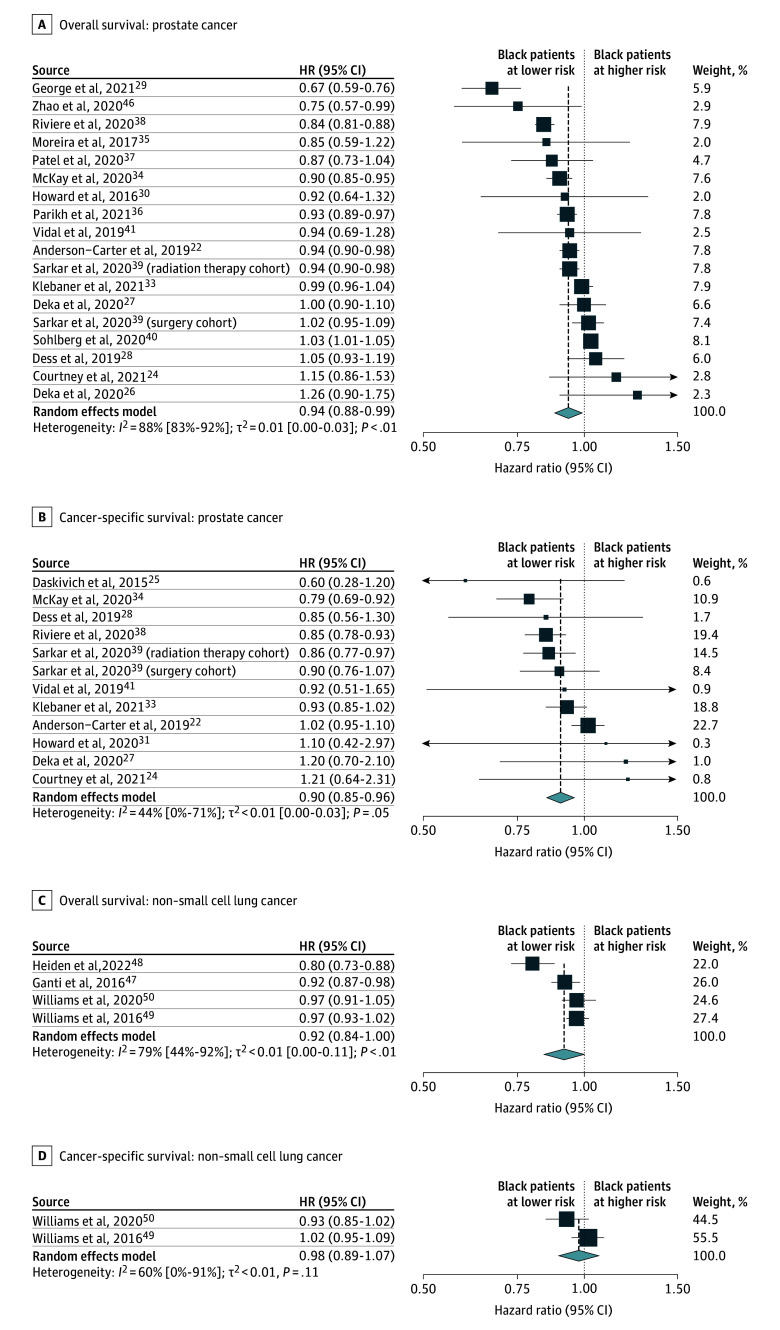
Forest Plots of Pooled Hazard Ratios (HRs) for Racial Differences in Survival Among Veterans With Prostate and Non–Small Cell Lung Cancer (NSCLC)

A total of 18 articles reported OS by race for veterans with prostate cancer, with HRs ranging from 0.67 (95% CI, 0.59-0.75) to 1.26 (95% CI, 0.90-1.75) (Figure 2A). OS was reported as not measurably different by race in 10 articles, with 7 reporting higher and 1 inferior OS for Black veterans. A total of 12 articles reported CSS by race for veterans with prostate cancer, with HRs ranging from 0.60 (95% CI, 0.28-1.20) to 1.21 (95% CI, 0.64-2.31) (Figure 2B). CSS was reported as not measurably different in 9 articles and higher for Black veterans in 3 articles.

### Non-Small Cell Lung Cancer

There were 4 articles reporting OS or CSS by race for veterans with early-stage or locally advanced NSCLC.^47,48,49,50^ All articles had a retrospective study design and analyzed data from a national cohort. Patients in these articles had been managed with either surgical, radiation, or systemic therapies. The articles evaluated survival outcomes associated with the following potential prognostic factors: clinical (race), tumor (stage), or treatment (surgery, radiation therapy).

A total of 4 articles reported OS by race for veterans with NSCLC, with an HR ranging from 0.80 (95% CI, 0.73-0.88) to 0.97 (95% CI, 0.93-1.02) (Figure 2C). OS was reported as not different in 2 articles and higher for Black veterans in 2 articles.

A total of 2 articles reported CSS for veterans with NSCLC by race with a HR ranging from 0.93 (95% CI, 0.85-1.02) to 1.02 (95% CI, 0.95-1.09) (Figure 2D). CSS was reported as not different in both articles.

### Pancreatic Cancer

There were 2 articles that reported OS by race for veterans with pancreatic cancer.^51,52^ Patients in these articles had non-metastatic disease at diagnosis and had been managed with upfront surgery. Both articles reported outcomes from a national VHA cohort evaluating the impact of socioeconomic factors, or the use of metformin, and did not find any measurable differences in OS by race.

### Head and Neck Cancer

There were 3 articles that reported OS or CSS by race for veterans with laryngeal or oropharyngeal cancers.^53,54,55^ Patients in these articles had been managed with either surgical, radiation, or systemic therapies. Two articles reported outcomes from a single VHA medical center evaluating the association of race, and the other compared outcomes from a national VHA cohort with population-level SEER data.

The 2 articles^53,54^ evaluating patients with laryngeal cancer reported similar OS between Black and White veterans. Similar CSS rates were also reported for Black and White patients in a national cohort of 9248 veterans with larynx cancer (20.3% Black). A single article evaluated veterans with oropharyngeal cancer and found similar OS rates for Black veterans in comparison with White veterans.

### Esophageal Cancer

One article was identified reporting OS by race for 122 veterans with esophageal cancer (of whom 25 [20.5%] were Black).^56^ This retrospective multisite study investigated clinical and tumor factors associated with early mortality. The authors divided their cohorts into early and late mortality groups and reported no differences by race in a univariate analysis; a multivariate analysis was not reported.

### Bladder Cancer

One article was identified reporting OS and CSS by race for 36 322 veterans with bladder cancer (3250 Black veterans [8.9%]).^57^ This retrospective study compared outcomes from a national VHA cohort to population-level SEER data. The analysis of veterans with bladder cancer showed similar OS and CSS rates for Black veterans in comparison with White veterans.

### Breast Cancer

One article was identified reporting survival by race among 8864 veterans with breast cancer (7336 female [82.8%]; 2166 Black [24.4%]).^58^ This retrospective study evaluated national VHA data to compare gender disparities. The authors reported that race was not associated with mortality based on available survival data (8589 patients).

### Multiple Myeloma

One article was identified reporting OS by race for 3807 veterans with multiple myeloma (883 Black veterans [23.3%]).^59^ This retrospective study used national VHA data to assess frailty in veterans with myeloma. The analysis demonstrated better OS rates for Black veterans in comparison with White veterans.

### All Cancer Types

One article was identified reporting OS and CSS for 4 485 170 veterans with any cancer (with 718 720 Black veterans [16.0%]).^18^ It compared the OS and CSS of veterans with the general US population stratified by race and ethnicity using the National Center for Health Statistics detailed mortality files. The analysis demonstrated fewer racial or ethnic disparities within the VHA and commented that “equal-access health care may partially address racial and ethnic mortality disparities, but other non–health care factors should also be explored.”

### Meta-Analysis of Survival

Of the 29 articles with sufficient data for the meta-analysis, 28 included an HR for OS by race and 17 for CSS by race. The random-effects model identified between-study heterogeneity was low to moderate.

**Figure 3.**
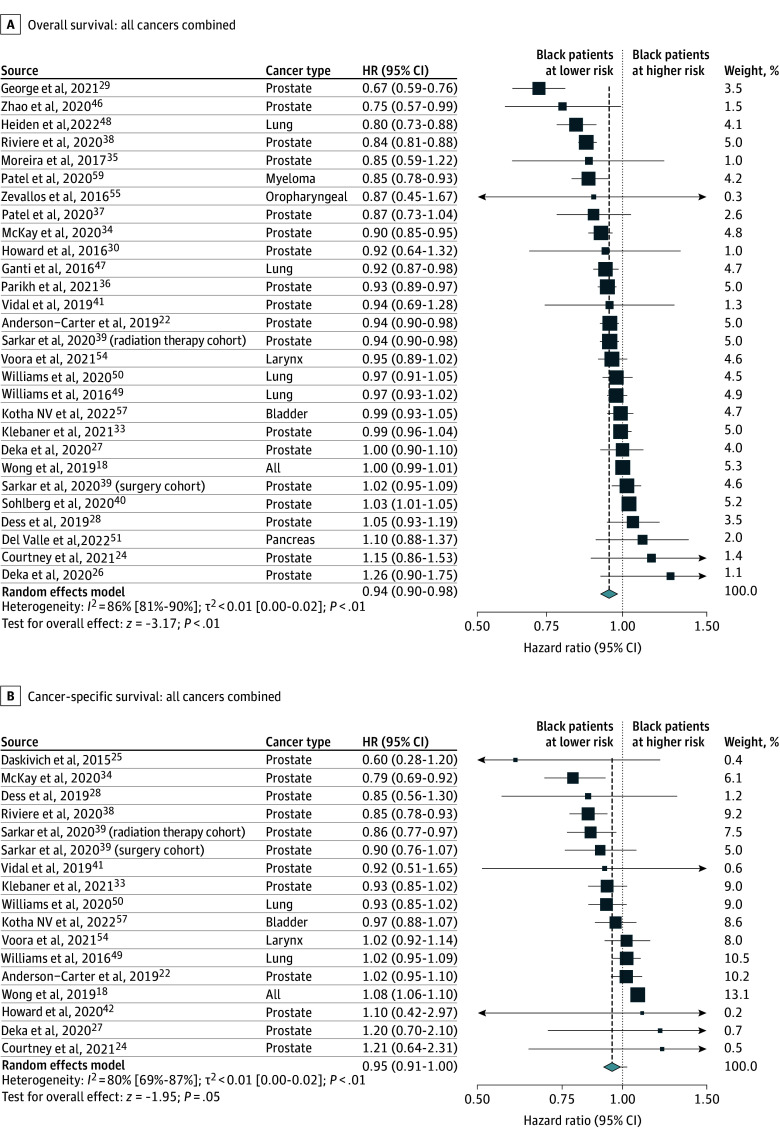
Forest Plots of Pooled Hazard Ratios (HRs) for Racial Differences in Survival Among All Cancer Types Combined

**Figure 4.**
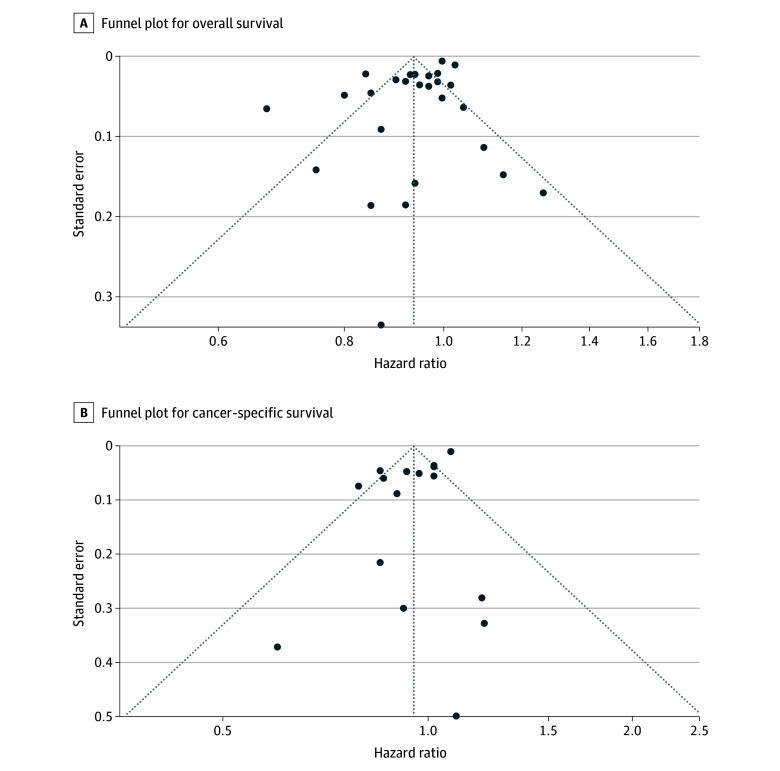
Funnel Plots Evaluating Publication Bias in Included Studies on Racial Differences in Survival Among Veterans With Cancer The distribution of studies around the pooled hazard ratio was approximately symmetrical, which indicates minimal evidence of publication bias.

The pooled HRs for OS and CSS were 0.93 (95% CI, 0.89-0.97) and 0.94 (95% CI, 0.90-0.98), respectively, indicating slightly improved outcomes for Black veterans across multiple cancer types (Figure 3). The funnel plots of OS and CSS were approximately distributed symmetrically, suggesting minimal publication bias (Figure 4).

The pooled HRs for OS and CSS among veterans with prostate cancer were 0.94 (95% CI, 0.88-0.99) and 0.90 (95% CI, 0.85-0.96), respectively, indicating slightly improved outcomes for Black veterans. Pooled HRs for OS and CSS among veterans with NSCLC were 0.92 (95% CI, 0.84-1.00) and 0.98 (95% CI, 0.89-1.07), respectively, indicating slightly improved outcomes for Black veterans.

## Discussion

This systematic review and meta-analysis analyzed the outcomes of over 600 000 veterans with cancer managed through the VHA. OS and CSS rates were comparable between Black and non-Black patients, and in some cases slightly better for Black patients. These findings contrast with persistent disparities and lower survival rates for Black patients with cancer observed in the broader US population.

The results suggest integrated health care systems might provide more equitable access to care and mitigate disparities in cancer outcomes. Specifically, among Black veterans with prostate cancer, the pooled analyses demonstrated slightly better survival compared with non-Black veterans, while outcomes among Black veterans with NSCLC were similar or better. These observations support the hypothesis that inherent patient differences by race may be less important in shaping outcomes disparities compared with access to care.

In the general US population, multiple structural factors contribute to the persistence of disproportionate access to care by race, including disparate levels of poverty and socioeconomic stressors,^60,61^ higher rates of underinsurance, health care system discrimination, and geographic barriers to high-quality care.^10,62^ Prior research has shown that Black individuals receiving care outside VHA are more likely to be screened at nonaccredited facilities, experience delays in diagnosis and treatment, receive care from lower-performing physicians,^7,10,63,64,65,66,67,68^ and are less likely to receive guideline-concordant therapy or participate in clinical trials.^63,69,70^

Meanwhile, VHA’s integrated care model is known to mitigate barriers to care through a single-payer structure with minimal financial burdens that offers colocated preventive, primary, specialty, and diagnostic services.^6,18,71,72,73^ In addition, ancillary services such as transportation support, housing assistance, and a national teleoncology program help improve outcomes that may attenuate differences by race for Black veterans who receive most of their care within the VHA.^18,74,75^

While VHA benefits reduce financial barriers to care, important sources of inequity remain for its eligible patients due to persistent structural factors that may be outside the scope of VHA policies, such as geographic residence and variation in facility utilization.^76^ It is important for future researchers to continue evaluating health care delivery systems’ impact on disparities, especially as it might influence additional factors along the pathway from structural inequity to survival. For example, it would be crucial to include an analysis of stage at diagnosis vs treatment, which would provide greater insights into whether integrated models of care are poised to mitigate cancer disparities on a broad scale, as opposed to conditionally benefiting only populations who are properly diagnosed and staged in a timely manner.

### Limitations

This study has several limitations. The data represent individuals selected into military service with eligibility for VHA benefits who have sustained engagement with the VHA that may differ by race. This raises the possibility that Black veterans included in these studies represent a positively selected subgroup with respect to health, resilience, or other unmeasured factors, a phenomenon that has been described as a “healthy Black veteran effect,” analogous to the healthy immigrant effect.^77,78^ Next, receipt of care within the VHA may introduce collider bias if both race and unmeasured health or socioeconomic factors influence inclusion in the analytic cohorts.^79^ Furthermore, the care received by veterans in these publications likely included care provided through community clinics outside the VA, when needed, that may have been distributed unequally by race. This introduces the possibility of associations toward the null or even in a protective direction independent of a true effect of the health care system itself.^80^

It deserves emphasis that the meta-analysis is dominated by prostate cancer studies. While differences in study time periods partially mitigate the risk of sample overlap, this limits generalizability across other cancer types.^81^ In addition, several leading causes of cancer death in the US, including colorectal, pancreatic, and breast cancers, were underrepresented or absent.

The potential confounding of geographic clustering must also be considered. Black veterans are disproportionately located in the Southern US, and a small number of VHA medical centers provide care for the majority of Black veterans with cancer.^73^ This concentration further limits the generalizability of the study’s findings.

Finally, as with all meta-analyses, the robustness of the analyses is contingent on the quality and completeness of the underlying studies, and heterogeneity across studies limits confidence for any pooled estimates. Some studies excluded large numbers of patients due to missing or incomplete data, introducing potential for selection bias. Decreased female representation and lack of stratification by sex also limit the ability to evaluate whether survival patterns are consistent among Black compared with non-Black women veterans.

## Conclusions

In this systematic review and meta-analysis, published data reporting the outcomes of veterans with cancer receiving care through the VHA demonstrate minimal differences in survival by race. These findings imply that integrated, comprehensive models of care that reduce financial and logistical barriers to care can mitigate disparities persistently observed in the broader US population. While this analysis cannot conclude that integrated health care systems, in and of themselves, can eliminate disparities in cancer care, it demonstrates the value of VHA’s health care delivery system for policymakers to look into further when evaluating strategies to further improve cancer outcomes across the US.

## Notes

Supplement. Data Sharing Statement
