# Supplementary material for: Equity and Cancer Survival Among Veterans Health Administration Patients: A Systematic Review and Meta-Analysis
Source: JAMA Netw Open. 2026 Jul 2;9(7):e2621585. doi: 10.1001/jamanetworkopen.2026.21585 (PMC13329710; doi:10.1001/jamanetworkopen.2026.21585)
Supplement: Supplement. — Data Sharing Statement [file jamanetwopen-e2621585-s001.pdf]

Data Sharing Statement

Bullard. Equity and Cancer Survival Among Veterans Health Administration Patients. *JAMA Netw Open*. Published July 02, 2026. doi:10.1001/jamanetworkopen.2026.21585

Data
Data available: No

Additional Information
Explanation for why data not available: The data underlying this article were derived from sources in the public domain and are incorporated in this article.
